# Sequencing Overview of Ewing Sarcoma: A Journey across Genomic, Epigenomic and Transcriptomic Landscapes

**DOI:** 10.3390/ijms160716176

**Published:** 2015-07-16

**Authors:** Laurens G. L. Sand, Karoly Szuhai, Pancras C. W. Hogendoorn

**Affiliations:** 1 Department of Pathology, Leiden University Medical Center, Leiden 2333 ZA, The Netherlands; E-Mail: L.G.L.Sand@lumc.nl; 2 Department of Molecular Cell Biology, Leiden University Medical Center, Leiden 2333 ZA, The Netherlands; E-Mail: k.szuhai@lumc.nl

**Keywords:** bone neoplasm, bone tumor, Ewing sarcoma, soft tissue tumor, targeted therapy, epigenetics, micro-environment, immunotherapy, next generation sequencing, splicing

## Abstract

Ewing sarcoma is an aggressive neoplasm occurring predominantly in adolescent Caucasians. At the genome level, a pathognomonic *EWSR1-ETS* translocation is present. The resulting fusion protein acts as a molecular driver in the tumor development and interferes, amongst others, with endogenous transcription and splicing. The Ewing sarcoma cell shows a poorly differentiated, stem-cell like phenotype. Consequently, the cellular origin of Ewing sarcoma is still a hot discussed topic. To further characterize Ewing sarcoma and to further elucidate the role of EWSR1-ETS fusion protein multiple genome, epigenome and transcriptome level studies were performed. In this review, the data from these studies were combined into a comprehensive overview. Presently, classical morphological predictive markers are used in the clinic and the therapy is dominantly based on systemic chemotherapy in combination with surgical interventions. Using sequencing, novel predictive markers and candidates for immuno- and targeted therapy were identified which were summarized in this review.

## 1. Introduction

Ewing sarcoma (EWS) is a high-grade sarcoma occurring predominantly in the bones of children and young adolescents, in which it is the third most common primary bone sarcoma, following osteosarcoma and chondrosarcoma. In adults, it occurs less frequently, but at this age, soft tissue and organ related involvement is more common [1,2]. At the cellular level, EWS has a poorly differentiated, stem cell-like phenotype with some degree of neurogenic features. These were partly represented by earlier classification as peripheral primitive neuroectodermal tumors (PNET). In the current World Health Organization (WHO) classification, however, PNET and a clinical variant of EWS known as Askin tumor, arising in the chest wall, are all classified as EWS based on the presence of a unifying pathognomonic chromosomal translocation [1]. This translocation forms a chimera gene fusing the *EWSR1* gene with a member of the ETS transcription factor family. Of the *EWSR1*-*ETS* translocations, *EWSR1*-*FLI1* is the most common with 85% of the cases. Other partners of *EWSR1* are *ERG* (10%), *ETV1*, *ETV4* and *FEV* [2]. No difference in survival was observed between the different translocation types [2]. There is an increasing body of evidence from tumors with histopathological appearance of EWS without the involvement of EWSR1 and/or ETS. The clinical relevance of this Ewing-like tumor family from classical EWS is yet unknown and is studied [3,4,5]. The incidence of EWS is three per million and around a nine-fold more in Caucasians compared to Africans [6]. A suggested genetic explanation for this is the presence of intronic Alu elements (retrotransposons) located near the breakpoint region. In the African population, an allele which lacks the majority of the Alu elements has been identified with an allele frequency of 8% [7]. Alu elements are potentially more preferred during recombination and their increase could increase the chance of a translocation to occur [8]. The lack of Alu repeats may contribute, but it cannot be the leading mechanism behind the observed difference in tumor incidence. Furthermore, a similar occurrence in Alu distribution was not observed in other *EWSR1* translocation positive sarcomas, like clear cell sarcoma [9]. A large genome-wide association study (GWAS) on EWS identified no single-nucleotide polymorphism (SNP) association at the *EWSR1* and *ETS* breakpoints. However, they did find three SNPs; rs9430161 on chromosome 1 upstream *TARDBP* (Tat activating regulatory DNA-binding protein), rs224278 on chromosome 10 upstream *EGR2* (early growth response 2) and rs4924410 at locus 15q15, which were associated with EWS with odds ratio of 2.2, 1.7 and 1.5, respectively. EGR2 is a target of EWSR1-FLI1 and TARDBP was proposed to be structurally and functionally similar to EWSR1 [10]. Further validation is required for the SNP at 15q15, since multiple genes are located in close proximity of it. The SNPs on chromosome 10 and 1 were more frequent present in Caucasians compared to Africans and could thereby be a factor in the differences in incidence of EWS in different racial patient populations [10]. Recently, another possible cause of the epidemiologic difference in the occurrence of EWS has been proposed. The EWSR1-ETS chimeric protein binds to GGAA microsatellites which differ in distribution between Caucasians and Africans. Caucasians have a higher frequency of repeats of 20–30 GGAA elements compared to Africans, which have a higher frequency of repeats longer than 30 elements. In a reporter gene assay, the highest EWSR1-FLI1 expression was observed when the GGAA microsatellite consisted of 20–30 motifs and this was concordant with the EWS target gene expression in relation to GGAA microsatellite length in EWS cell lines [11,12]. This suggests that the expression inducible capability of EWSR1-ETS can be larger in Caucasians compared to Africans.

## 2. Relation between EWSR1-ETS and the Cell of Origin of EWS

There is an ongoing debate on the identification of the cell of origin of EWS. Expression of the fusion protein leads to more stem cell-like phenotypes and expressions of neuro-ectodermal markers [13]. In addition, *EWSR1* is expressed in many tissues, its function is poorly understood and the *EWSR1* gene is involved in translocations in multiple other tumors [14,15,16,17]. Multiple cells of origin have been suggested, such as mesenchymal stem and neural crest cells [13,18,19]. In order to shed some light on this debate, the effect of induced expression of the chimeric protein in non-tumorigenic cells was investigated. It was expected that the translocation had a large impact on cell homeostasis and interfered at multiple levels in endogenous processes. To study the impact of this gene chimera, primary human fibroblasts were transfected with an EWSR1-FLI1 construct and that led to a TP53 dependent growth arrest. This points towards the need of additional (secondary) changes to be able to transform [20]. Likewise, in other studies, which used *EWSR1-ETS* transfected adult human mesenchymal stem cells (MSCs), an additional mutation was needed for the cells to form tumors; while transformation was possible using unmodified pediatric MSCs [18,21,22]. Animal models containing inducible *EWSR1-FLI1* constructs led to phenotypically varying tumors from malignant peripheral nerve sheath tumors to myeloid/erythroid leukemia [23,24]. These observations directed towards a hypothesis that certain epigenetic changes might be needed to result in an EWSR1-ETS driven tumor and that this partly dictates the phenotype of the tumor. The presently hypothesized cells of origin are MSCs and neural crest cells. This is based on their capability to endure expression of *EWSR1-ETS* gene chimera without additional mutations, and the finding that transient *EWSR1-ETS* expression leads to a tumor similar to EWS at the level of expressed cellular markers and micro-array expression data [22,25]. Recently, a new mouse model has been created to mimic EWS using specific selected cells of the embryonic superficial zone of the long bones. In these animals, EWS-like tumors developed without any additional gene modifications. This might be a leap forward in creating a mouse model for EWS [19]. To gain further insight into the tumor specific genetic changes multiple massive parallel sequencing studies were performed at the genome, the transcriptome and the epigenome level (Table 1). By combining the results of these studies, researchers may identify landscape marks in the EWS OMIC atlas explaining some of the mechanisms behind the behavior of Ewing sarcoma with the aim to identify new, targeted therapeutic targets These targets can be validated by combining functional studies and testing In addition, this might shed light on the cell of origin and secondary events necessary for tumor formation and changes that are related to a more therapy resistant or more aggressive phenotype.

**Table 1:** Next generation sequencing studies in Ewing sarcoma.

Sequnce Level	Study	Method	Platform	Material	Data Accessibility	
**Genome**	Brohl *et al.* [26]	whole genome paired-end sequencing	Complete genomics	6 germline control paired samples	not accessible	
		targeted genomic sequencing	Iontorrent	65 tumor samples and 36 cell lines	supplementary	(only mutations)
	Tirode *et al.* [27]	whole genome paired-end sequencing	Illumina Hiseq2000	112 germline control tumor samples	EGAS00001000855	raw data of 200 samples
					EGAS00001000839	raw data of 38 samples
	Crompton *et al.* [28]	whole genome paired-end sequencing	Illumina Hiseq2000	7 germline control tumor samples	supplementary	(analyzed data)
		whole exome paired-end sequencing	Illumina Hiseq2000	26 germline control tumor samples, 66 tumor samples, 4 paired relapses, 11 cell lines	supplementary	(analyzed data)
	Lawrence *et al.* [29]	whole exome sequencing	Illumina Hiseq2000	20 germline control tumor samples	not accessible	
	Jiang *et al.* [30]	targeted exome sequencing	FoundationOne platform	28 tumor samples	supplementary	(only mutations)
**Epigenome**	Riggi *et al.* [31]	H3K27ac ChIP-seq	Illumina Hiseq2000	A673, SKNMC cell line, pediatric mesenchymal stem cells and 4 primary tumor samples	GSE61944	raw data
		H3K4me3 ChIP-seq				
		H3K27me3 ChIP-seq				
		FLI1 ChIP-seq		A673, SKNMC cell line		
		p300 ChIP-seq				
		GABPA ChIP-seq				
		ELF1 ChIP-seq				
		WDR5 ChIP-seq		A673, SKNMC cell line and pediatric mesenchymal stem cells		
		ATAC-seq	Illumina Hiseq2500	SKNMC cell line and mesenchymal stem cell	GSE61951	raw data
	Tomazou *et al.* [32]	DNA methylation RRBS	Illumina Hiseq2000	A673 cell line	tomazou2015 website	raw and analyzed data
		DNA methylation WGBS				
		ATAC-seq				
		H3K4me3 ChIP-seq				
		H3K27me3 ChIP-seq				
		H3K27ac ChIP-seq				
		H3K56ac ChIP-seq				
		H3K9me3 ChIP-seq				
		H3K4me1 ChIP-seq				
		H3K36me3 ChIP-seq				
	ENCODE [ 33]	RRBS	ND	SKNMC cell line	ENCSR000DDT	raw and analyzed data
		FOXP2 ChIP-seq			ENCSR000BGB	raw and analyzed data
		POLR2AphosphoS5 ChIP-seq			ENCSR000BPL	raw and analyzed data
		H3K4me3 ChIP-seq			ENCSR000DXL	raw and analyzed data
	Guillon *et al.* [34]	FLI1 ChIP-seq	Illumina 1G	A673, SKNMC cell line	not accessible	
	Bilke *et al.* [35]	FLI1 ChIP-seq	Illumina genome analyzer I	A673 cell line	GSE27524	raw data
		-				
		E2F3 ChIP-seq				
	Wei *et al*. [36]	FLI1 ChIP-seq	Illumina genome analyzer	SKNMC cell line	SRP002475	raw data
		ERG ChIP-seq		CADO-ES1 cell line		
**Transcriptome**	Brohl *et al.* [26]	whole transcriptome TruSeq paired-end sequencing	Illumina Hiseq2000	31 cell lines and 58 tumor samples	not accessible	
	Crompton *et al.* [28]	whole transcriptome TruSeq paired-end sequencing	Illumina Hiseq2000	20 tumor samples, 3 paired relapses, 9 cell lines	supplementary	analyzed data
	Sankar *et al.* [37]	whole transcriptome TruSeq single-end sequencing	Illumina Hiseq2000	A673 and TTC-466 cell line	supplementary data	analyzed data
					SRA096343	raw data
					SRA096347	raw data
					SRA096354	raw data
	Marques Howarth *et al.* [38]	3ʹ SEQ	Illumina Genome Analyzer II	pediatric multipotent cells	GSE60891	raw data
		whole transcriptome TruSeq paired-end sequencing	Illumina Hiseq2000	A673 cell line	GSE60949	raw data
	Riggi *et al.* [31]	whole transcriptome sequencing	Illumina Hiseq2000	A673, SKNMC cell line	GSE61950	raw data
	Tomazou *et al.* [32]	whole transcriptome TruSeq sequencing	Illumina Hiseq2000	A673 cell line	tomazou2015 website	raw and analyzed data
	Selvanathan *et al.* [39]	whole transcriptome paired-end sequencing	Illumina Hiseq2000	7 cell lines	not accessible	
		FLI1 CLIP-seq	Illumina Hiseq2000	TC32 cell line		
	Erkizan *et al.* [40]	BruDRB-seq	Illumina Hiseq2000	TC32 cell line	not accessible	
		RIP-seq	Otogenetics			

## 3. Genome Map

To identify possible secondary genetic and genomic alterations related to the development of EWS and its biology, several groups performed genome-wide studies such as: whole genome sequencing (WGS), whole exome sequencing (WES) and whole transcriptome sequencing (WTS) [26,27,28]. These three types of studies included WGS of 123 tumor samples in parallel with the normal tissue derived germline controls, WES of 92 tumors of which 26 with paired normal control and 11 cell lines and WTS of 92 tumors and 42 cell lines resulting in data about structural rearrangements and variations, somatic mutations and expression profiles.

For a long time, EWS was known as a genetically stable tumor with rarely occurring additional mutations. Only a few genomic changes such as *TP53* mutations or *CDKN2A/CDKN2B* deletions were observed in a minority of samples in retrospective studies and they were reported to be associated with an inferior outcome in a multivariate analysis [41,42]. The search for secondary mutations that provide a permissive genetic background, and might explain how the EWSR1-ETS chimera protein transforms cells, remained unsuccessful for over twenty years after the initial identification of the *EWSR1-FLI1* fusion gene [43]. The goal of the genome sequencing studies was to identify the missing link in this area. Both WGS and WES studies detected only a very low number of somatic mutations (0.65–0.15 per Mb) although different statistics for analysis were used [27,28]. Similarly, the low number of single nucleotide variations (SNV) in EWS has been reported in an earlier study and was, when compared to other tumors, one of the lowest [29]. Possible causes for the low number of SNVs could be related to the pathognomonic gene fusion acting as a direct tumor driver, and to the young age of onset of the tumor with possible fewer gained environmental mutations. Rhabdomyosarcoma (RMS) consists of both fusion gene positive and negative subtypes and the fusion positive subtype contained significant less mutations compared to the fusion negative subtype [44]. The number of mutations detected in fusion positive RMS was similar to EWS. The number of additional mutations correlated with age in both the RMS and EWS, confirming an age related factor [28,44]. Another retrospective study confirmed that the increased number of somatic mutations was in a univariate analysis correlated to shorter survival time [27]. This might partly explain why an increased age is correlated with inferior prognosis in EWS, but it could also be due decreased tolerance to chemotherapy [45,46]. In biopsies, the most common kind of mutation detected was a C to T transition, which was linked to the common event of deamination of methylated cytosines [28]. The number of mutations was, as expected, increased in post-chemotherapy samples and an association between the increased numbers of novel mutations with a poor patient outcome was observed [28]. In theory, these clones might already have been present but remained undetected due to tumor heterogeneity. Alternatively, these mutations were caused by the treatment resulting in a drug resistance phenotype. This would be very interesting for understanding treatment response prediction. Overall, EWS is from a global genomic perspective a relatively stable tumor with low number of somatic mutations, implying a functional mutation recognition and repair mechanism.

### 3.1. Structural and Copy Number Variant Map

All *bona fide* EWS contained an *EWSR1-ETS* translocation and these were detected in all tumors and cell lines tested [26,27,28]*.* In the study by Brohl *et al*. [26], however, seven cases were identified with cellular phenotype similarly to EWS but without an *EWSR1-ETS* translocation, supported by the fact that these samples cluster separately based on RNA expression profile. This observation supports the notion of the existence of a Ewing-like tumor with clinical- and histo-morphological appearances similarly to EWS but carrying other, specific translocations such as, *BCOR-CCNB3*, *EWSR1-NFATc2*, *FUS-NFATc2* and *CIC-FOXO4* and *CIC-DUX4* [3,4,5]. As these entities are rare, follow-up studies have to show if these groups should be further stratified based on the genes involved or might be lumped as one clinical entity, Ewing-like sarcoma. None of the sequenced EWS samples detected an additional, commonly occurring translocation co-existing with *EWSR1-ETS*.

Although EWS tumors with a complex karyotype occur in a minority of cases, there are some common chromosomal alterations. These are gain of chromosome 1q, 8, 12 and loss of 9p21 and 16q [47,48,49,50]. Gain of chromosome 1q and chromosome 16q loss were strongly co-associated caused by an unbalanced translocation der(16)t(1;16) [47,51,52,53]. The frequency of 1q gain was, in various studies, associated with a dismal prognosis and was higher in chemotherapeutic treated tumors [27,28,47,48,54,55]. The responsible factor for this association was investigated by Mackintosh *et al.* who compared samples with and without 1q gain and 16 loss. At chromosome 1q, they identified increased expression of the gene Cell Division Cycle Protein 2 (*CTD2*), also known as Denticleless E3 Ubiquitin Protein Ligase Homolog (*DTL*), as the suspected factor [48]. DTL is, like TP53, involved in DNA damage repair and could therefore have an effect on tumor progression [56]. The chromosome 1q gain is not a EWS specific aberration, as it is one of the most frequently observed secondary changes in many tumor entities and even in cultured embryonic stem cells [57]. The large heterochromatic regions at 1q12 might be responsible for the frequent translocation breakpoint leading to gain of the long arm of chromosome 1 [58,59,60]. As was observed with 1q, gain of chromosome 8 and 12 was present in many other tumors summarized by the progenetix website [61]. According to this website, these chromosome gains might be linked to pluripotency and proliferation. Chromosome 12 gain has also been observed in cultured human embryonic stem cells [62]. The oncogene associated with the increased tumorigenity for chromosome 12 gain is not clear since next to *NANOG* it contains many genes including known oncogenes *CDK4, ERBB3, GLI1* and *MDM2.* For chromosome 8 gain, the increased expression of the oncogene *MYC may be* the attributing factor; however, EWS without the gain of chromosome 8 show similarly high expression of *MYC* [63,64,65,66]. Homozygous loss of 9p21 is, with about 12%, less common in EWS but could have a large impact since a well-known cell cycle regulator *CDKN2A/CDKN2B* is in this locus. Huang *et al.* [41] demonstrated in a retrospective study of 60 patients that the loss of *CDKN2A/CDKN2B* has a negative effect on the overall survival. Recently, Tirode *et al.* [27] analyzed 300 EWS samples and did not observe a significant difference in overall survival of patients with or without *CDKN2A/CDKN2B* loss. This underlines the importance of large sample size in studies of EWS, when trying to predict the effect of genomic alternations on prognosis. However, no data on chemotherapeutic response was presented of these patients, which has been reported to be significantly worse in patients with a *CDKN2A/CDKN2B* loss [27]. Loss of heterozygosity (LOH) is detected in earlier studies in a minority of the patients and was investigated using micro-satellite instability markers and identification markers, but no overlapping chromosomal regions were detected [67,68]. A recent study examined LOH in only six EWS samples by using SNP microarrays and showed some overlapping chromosomal regions with the ones reported earlier [69]. These were 17p and 11p and may be relevant to verify since *TP53* is located at the 17p chromosomal region. In several tumors inactivation of *TP53* has been reported due to point mutations or, less frequently, homozygous deletion, or deletion in combination with point mutation due to LOH. Intriguingly, in EWS inactivation of *TP53* caused by deletion was found as a rarely occurring event in earlier studies and was not even reported in any of recent large genomic landscape studies [26,27,28,41,48,49,70].

In the recent NGS studies, copy number alterations were detected in EWS but no common alteration was found. The copy number and structural alterations may merely represent secondary changes leading to a complex karyotype, which was found to be negatively associated with survival in earlier studies and was confirmed by genome sequencing study of Tirode *et al.* all retrospectively [27,47,71].

### 3.2. Mutation Map

**Figure 1:**
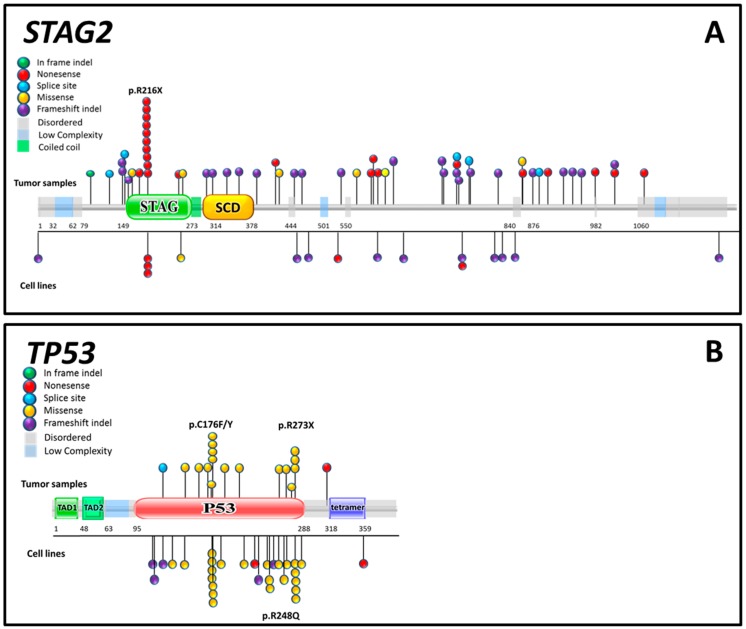
Mutation overview of reported *STAG2* and *TP53* in Ewing sarcoma. Overview of published mutations on *STAG2* and *TP53* from next generation sequencing data divided in five mutation subtypes based on data collected from 472 tumors and 54 cell lines. (**A**) Overview of the *STAG2* mutations (**B**) Overview of the *TP53* mutations. Amino acid sequence of the proteins is presented with different protein domains annotated in boxes and every sphere represents a reported mutation.

Although EWS contains few SNVs, their distribution over the genome is quite specific. The most commonly affected genes found in the genomic landscape studies were *STAG2* and *TP53* with an occurrence in patients of respectively 9%–21.5% and 5.2% to 7% and both were in a retrospective study in a univariate analysis associated with poor prognosis [26,27,72]. The most commonly mutated gene *STAG2* was only recently reported for the first time in EWS [73]. The distribution of the mutations is striking, with a quarter of the cases having a mutation at R216X, which is a possible CpG site and might be linked to a STAG2-DNA methylation pattern (see Figure 1A). The mutated *STAG2* status correlated only with an increase in structural variants and no other of the tested parameters [27,28]. This observation may be related to the function of STAG2, as it is a subunit of the cohesin complex and involved in chromatin modeling, chromatin cohesion, repair of stalled replication forks and double-strand breaks (DSBs) [74,75,76,77]. *STAG2* or other mutations in the cohesion complex were observed also in other tumors, including glioblastoma, myeloid malignancies, colon cancer and bladder cancer [72,73,78,79]. In colon cancer and glioblastoma, cohesin complex mutations were associated, like in EWS, with an increase in structural variants and aneuploidy [73,79]. In contrast, in myeloid malignancies this was not observed and in bladder cancer an inverse association was reported [80,81]. However, in myeloid malignancies, like in EWS, cohesin mutations were associated with poor prognosis [78]. In addition, when one of the cohesin complex genes was mutated in myeloid leukemia cell lines, less cohesin was bound to the chromatin [80]. Since cohesin is a key regulator of the chromatin structure and consequently influences gene expression, a reduction in the cohesin bound to the chromatin could affect the global gene expression [74,80,82]. *TP53* is the second most common mutated gene in EWS and is one of the most common mutated genes in all tumors [83]. The frequency of *TP53* mutations is slightly lower compared to earlier reports with an average of 10%. The two most frequent detected *TP53* mutations were the p.C176F and p.R273X of which p.R273X has been reported earlier [84]. In the International Agency for Research on Cancer (IARC) database, p.R273X is, like in EWS, a hot spot mutation. Yet, the most frequent *TP53* mutation in EWS p.C176F is remarkably not listed as a hot spot in the IARC database. In addition, the IARC database hot spot mutation p.R248Q is detected in only one tumor sample and only in one cell line, although it has been reported more frequent in earlier studies. This suggests that more samples are needed for a clear *TP53* mutation pattern (see Figure 1B) [26,27,28,83,85]. Mutations of *STAG2* and *TP53* showed a trend for co-occurrence with a synergistic negative effect on prognosis when both mutations were present. They are both involved in the checkpoint and repair processes, which may be further abrogated when both genes are mutated [27]. A trend for mutual exclusivity of TP53 mutation and the loss of CDKN2A with only a few exceptions were present. Moreover, CDKN2A loss and STAG2 mutation were mutual exclusive [27,28]. This indicates that CDKN2A and STAG2 may be involved in complementary essential processes such as cell cycle and chromatin remodeling. Having a mutation in both genes may be lethal or redundant for EWS tumors [27,28,41,42,86,87]. To correct errors that may be caused by the relatively low numbers of cases analyzed, validation of these data in a bigger study is necessary.

Other somatic gene mutations in EWS, described in three large genomic studies, were low and not recurrent. All three studies reported a different process to be most influenced by these somatic mutations. Tirode *et al.* [27] found mutations in several epigenetic regulators with *EZH2* as the most frequent mutated gene (3/112 cases), whereas Crompton *et al.* [28] reported mutations in other ETS transcription factors, including *ERF* (3/46 cases). Brohl *et al.* [26] reported mutations in the DNA repair pathway, in specific, with the deleterious polymorphism K3326X in *BRCA2* (4/55 cases) and a mutation in *RAD51* (1/55 case). An earlier study identified only four mutations in 75 EWS tumors with a hotspot array of 275 recurrent mutations across 29 genes which were not reported by these large genomic studies [88]. A recent study in chemotherapy-treated EWS tumors observed mutations which had implications for further targeted therapy response, such as KRAS [30].

Genome-wide sequencing of EWS was expected to show a common secondary event that would help to understand and model Ewing sarcoma and its onset. However, no common secondary event was identified. Overall, EWS was found to be a relatively stable tumor with a low frequency of mutations, which were scattered across the genome and acted dominantly on cell cycle processes. This suggests that these mutations occur during tumor progression and may be used as a marker for tumor progression but are not associated with the onset of EWS. Consequently, this may indicate the involvement of other factors in the onset of EWS pointing to disturbances at the epigenetic level as potential candidate.

## 4. Epigenome Map

**Figure 2:**
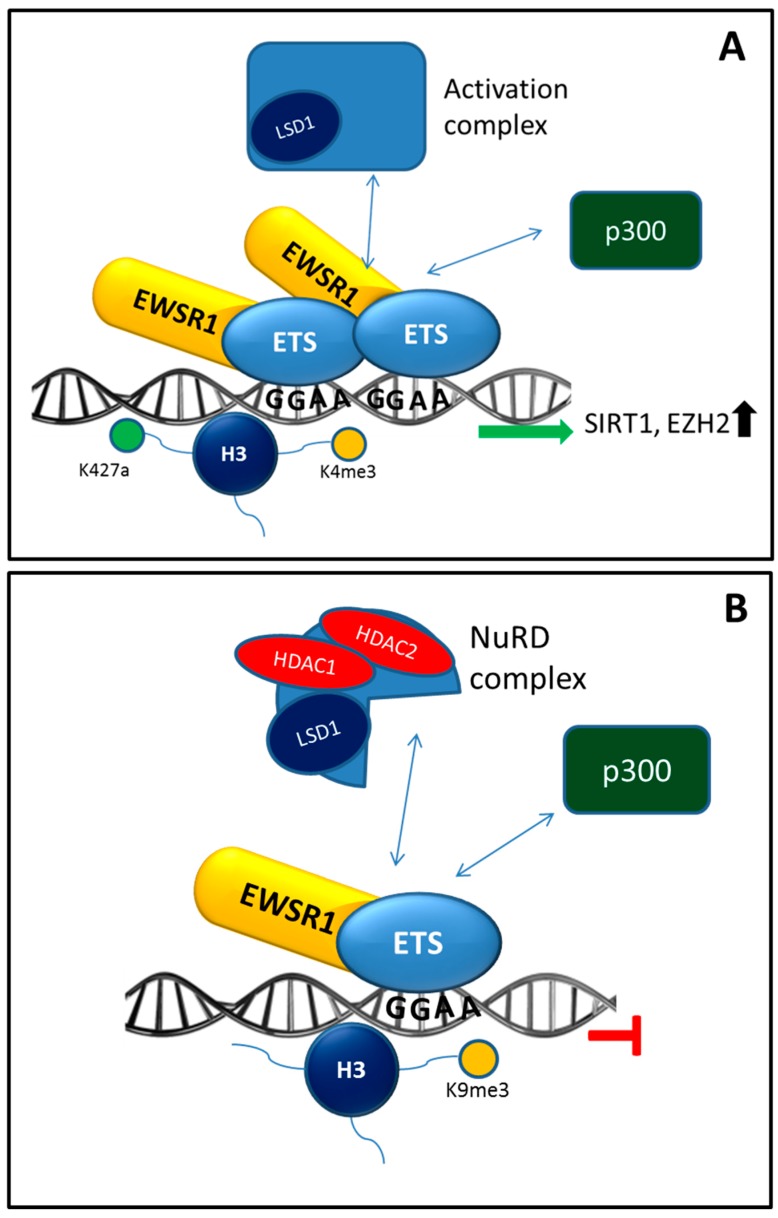
EWSR1-ETS mediated epigenetic activation and repression of gene expression. Possible mechanisms of how EWSR1-ETS acts as a transcription activator or repressor with different chromatin remodelers and is associated with different epigenetic histone modifications. (**A**) EWSR1-ETS activation complex binds to GGAA microsatellites. The complex attracts LSD1 in a yet unidentified activation complex and p300, which is needed for efficient transcription. The activation complex may bind to H3K4me3 and H3K27ac histone marks which, in turn, may lead to upregulation of the epigenetic modifiers *SIRT1* and *EZH2*; (**B**) EWSR1-ETS repression complex binds to single GGAA elements and scavenges for p300, but, as it is insufficient to create an activating complex, it may recruit NuRD repression complex which may lead to further repressed expression. In addition, these repression sites are marked with H3K9me3 histone mark.

Epigenetic modification involves both histone and DNA modifications such as acetylation or methylation of histone proteins and methylation of CpG islands. The DNA accessibility for transcription factors and polymerases, and thereby transcription, is partly regulated by these modifications. Classical sequencing reactions are not suited for the detection of epigenetic changes, therefore additional treatments have to be applied to detect these modifications. Examples of treatments to detect DNA methylation are MeDIP-seq, methylated DNA immunoprecipitation sequencing and WGBS, whole-genome bisulfite sequencing [89,90]. More complex approaches should be used to detect modifications influencing histone composition, such as ChIP-seq, chromatin immunoprecipitation sequencing; ChIP-exo, chromatin immunoprecipitation-exonuclease, or the detection of DNase-I sensitive sites [31,91,92]. As these approaches are complex reactions and not uniformly applied in different laboratories, comprehensive epigenome mapping of tumors are rarely published, although the ENCODE project, specifically set up for this, has generated a general overview [31,33,91,92]. Many parts of the epigenome in tumors however have been reported, since it is thought to have great therapeutic potential [93,94,95,96]. Recently an epigenome overview has been published by Tomazou *et al.* [32] covering the epigenome and transcriptome of EWS cell line A673 with inducible EWSR1-FLI1 knockdown construct. Four separate clusters of histone marks were detected with different effects upon knockdown of EWSR1-FLI1 [32]. Furthermore unique EWS open chromatin structures at distant enhancer and super-enhancers sites were detected, suggesting an important role for epigenomic regulation [32]. This might be related to the earlier described binding of the EWSR1-ETS fusion protein to GGAA containing microsatellite elements at enhancer sites and thereby affecting expression of downstream located genes (see Figure 2A) [31,97,98]. However, experimental evidence is lacking here. Binding to GGAA elements is an ETS specific effect and acts specifically on genes which do not contain a TATA box promoter [99]. Examples of such genes are *CAV1*, *NR0B1* and *FCGRT.* The binding of EWSR1-FLI1 to GGAA microsatellitesmight lead to multimer formation which is needed to attract sufficient number of chromatin remodelers necessary for the sustained expression [31,34,98]. An important attracted chromatin remodeler for this sustained expression is p300 that acetylates histone 3 lysine H3K27 (H3K27ac). Monomeric EWSR1-FLI1 binding to a single GGAA element could not activate transcription and even inhibited gene expression, marked by the H3K9me3 histone modification (see Figure 2B) [32].This might be due to insufficient attraction and binding of p300 since the fusion protein lacks a p300 binding site while wild-type ETS transcription with p300 binding sites could attract p300 and activate transcription [31,100]. In pediatric mesenchymal stem cells, induction of EWSR1-FLI1 led to a histone pattern at the EWSR1-FLI1 bound GGAA microsatellites which was similar to the pattern in EWS cell lines. Inhibition of EWSR1-FLI1 led to a decrease in activation of histone mark H3K27ac, which supports an active role of EWSR1-FLI1 in chromatin remodeling [31,32]. The H3K27 acetylation was especially associated with EWSR1-FLI1 bound enhancers [32]. It has to be noted that the overlap of ChIP-seq detected EWSR1-ETS binding sites was low with only 21% between *EWSR1-FLI1* carrying cell lines and 17.2% between *EWSR1-FLI1* and *EWSR1-ERG* carrying cell lines [31,36]. If these are all cell culture related artifacts or are due to accessibility of the DNA is not known. Another chromatin remodeling complex bound by EWSR1-FLI1 is the NuRD complex containing HDAC2 and HDAC3 proteins. These HDACs, when together with CHD4, can be active in the NuRD complex. Consequently, binding of the NuRD complex to EWSR1-FLI1 leads to repression of gene expression [101]. EWSR1-FLI1 regulated repression of expression was reverted by HDAC inhibitors and inhibiting histone demethylase LSD1, another NuRD complex protein. The NuRD complex is involved in many processes, especially in blood vessel development and integrity [32,102,103,104]. The interaction of EWSR1-ETS with the epigenetic remodelers is further increased by binding of EWSR1-ETS to the promotor of enhancer of zeste homolog 2 (*EZH2*) and Sirtuin 1 (SIRT1), thereby upregulating this histone methyltransferase and deacetylase [105,106]. The EZH2 mediated effect in the cell was dependent on HDAC activity, demonstrating a cross interaction between two EWSR1-ETS modulated chromatin remodelers [105]. Overall, a complex interaction between EWSR1-ETS, chromatin and chromatin remodelers is needed in Ewing sarcoma to execute its oncogenic effect. As described earlier, transient expression of EWSR1-ETS in cells from different origin resulted in different phenotypes. This might be, in part, attributed to the chromatin state near GGAA microsatellites. An open chromatin structure at the enhancer and super-enhancer sites, as identified by Tomazou *et al.* [32], may be needed for the transforming effect of a EWSR1-ETS fusion protein in the development of Ewing sarcoma and if a more closed chromatin state was present an *EWSR1-ETS* translocation would lead to different effects or cell death. [32]. Although this is an attractive and plausible hypothesis, there is no experimental evidence yet to support this notion. The microenvironment, through for example, proliferative signaling, could greatly influence the chromatin state and have an interplay between EWSR1-ETS oncogenic properties. The other way around, tumor cell induced signaling can change the differentiation status of cells allocated in the tumor and distant microenvironment.

The type of mutations identified in EWS tumors pointed towards presence of methylated CpG sites, as mentioned in the genome map chapter. DNA methylation in EWS is studied only in a limited number of studies that used various techniques. In a recent relative small retrospective study by Park *et al.* [107], it was shown that patients with a poor outcome had increased methylation of CpG islands compared to patients with a better outcome, although the total hypermethylated genes was limited with only 10% of the investigated genes [107]. Their observation showed a similar proportion of genes with methylated CpG islands to an earlier study on DNA methylation using a different methylation micro-array [108]. Although the proportions were similar, the majority of the actual detected genes identified were different, having only six genes in common (*LYN*, *EPHA3*, *ESR1*, *MAP3K1*, *NGFR* and *SOX17*) in two studies. Compared to clear cell sarcoma and rhabdoid tumor of the kidney the same low number of hypermethylation of CpG islands was observed, but the number of significant hypomethylated genes was similar [109]. Since this study contained only four Ewing sarcoma samples, a larger study with more samples using the same platform should be performed. Whole genome DNA methylation was also performed in the earlier mentioned epigenome-wide study of Tomazou *et al.* [32]. Through WGBS, they observed less DNA methylation at actively expressed genes compared to non-expressed genes, suggesting an involvement of DNA methylation in the *EWSR1-ETS* mediated gene expression effect. However knockdown of *EWSR1-ETS* did not change the DNA methylation pattern. An alternative method to investigate the DNA methylation would be by using the PACBIO RSII sequencer system (Pacific Biosciences, Menlo Park, CA, USA). This system can detect the methylated CpG sites during the sequencing process and, as it does not need any amplification or chemical modification step, it has no probe bias. An unbiased sequencing approach could help to identify DNA methylation pattern in primary tumors and see if the pattern is the same in EWS tumors compared to cell lines. Since cell lines are used as models for EWS tumors and DNA methylation at whole genome level is only studied in cell lines.

## 5. Transcriptome Map

**Figure 3:**
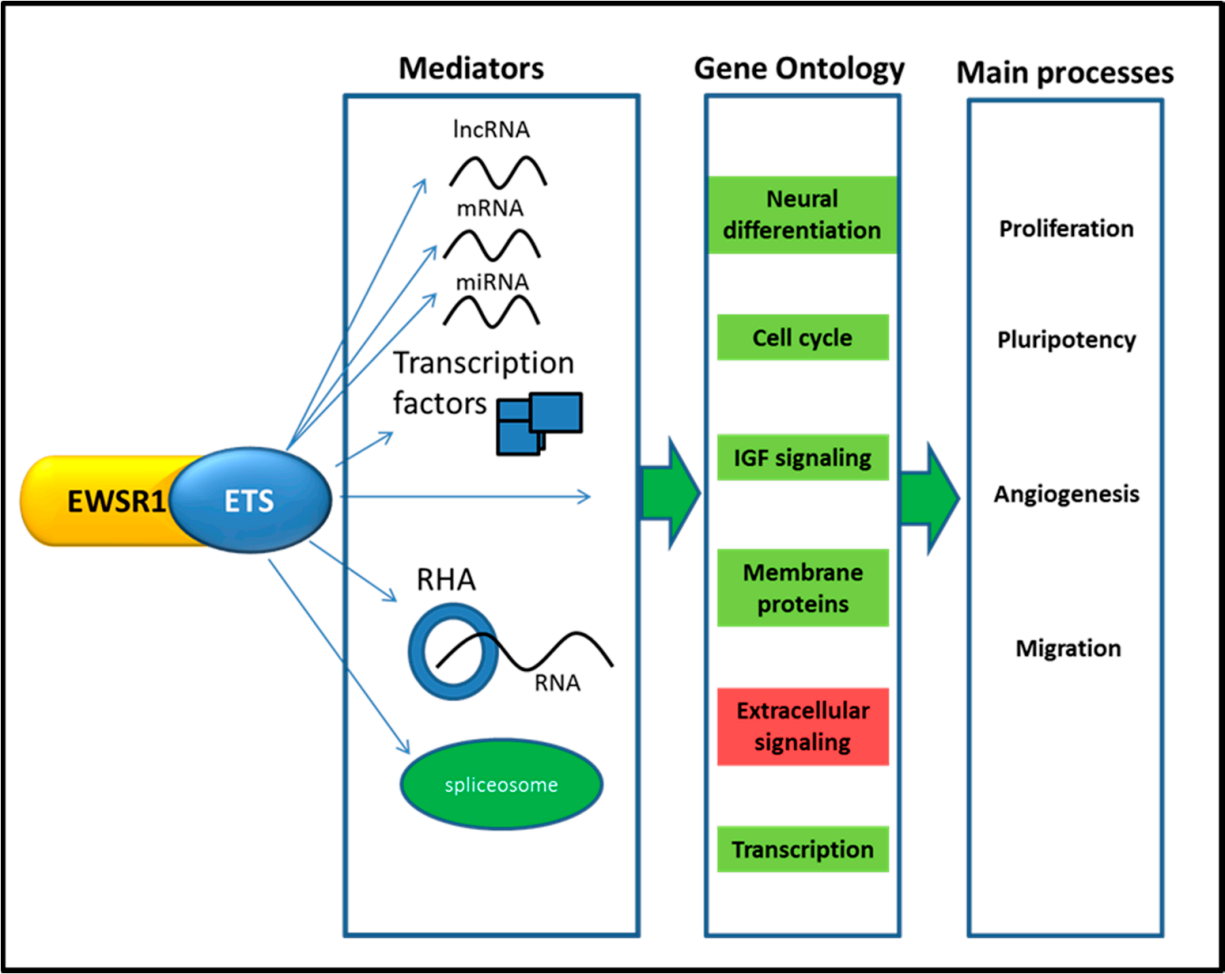
The influence of EWSR1-ETS fusion protein at the transcriptome level. EWSR1-ETS fusion protein acts as an aberrant transcription factor that influences the regulation of mRNA, lncRNA and miRNA expression levels. In addition, by binding to RHA additional transcripts can be bound and this might interfere with the stability of these transcripts. Alterations in epigenetic activity lead to up- and downregulation of a number of transcription factors and thereby interfere indirectly with gene expression. Furthermore, EWSR1-ETS fusion protein binds to the spliceosome and thereby altering splicing processes. By acting on these mediators, multiple cellular pathways are affected. The summarizing gene ontology clusters of the upregulated (green) cellular pathways are cell cycle, membrane proteins, IGF signaling and transcription and the downregulated is extracellular signaling (red). The main processes influenced by these gene ontology clusters are an increase in proliferation, pluripotency, migration and angiogenesis.

An EWSR1-ETS rearrangement affects gene expression levels, as mentioned above. In addition, it affects the expression of non-coding RNAs and splicing of RNAs by binding to the polymerase II complex protein hsRPB7 and to RNA helicase A (RHA) (see Figure 3) [40,110,111]. The effect of EWSR1-ETS on gene expression levels has been investigated with microarrays and studied in cell and animal models [19,20,22,112]. A meta-analysis of earlier micro-array studies was performed and compared the expression levels of other sarcomas demonstrating a specific EWS signature [113]. Knockdown studies of the most common fusion protein EWSR1-FLI1 revealed that it causes both downregulation and upregulation of numerous genes involved in extracellular and intracellular processes [35,114]. Downregulated genes were involved in extracellular signaling and signaling regulation, including multiple chemokines and interleukins (such as, CXCL8, CCL2 and IL1A) [38,101]. Upregulated genes were involved in neural differentiation, transcription and cell cycle and included membrane proteins [114,115,116,117]. Examples of external validated membrane proteins upregulated by EWSR1-ETS fusion protein are *STEAP1*, *GPR64*, *CD99*, *CAV1 and CHM1* [116,118,119,120,121]. These membrane proteins are interacting with the surrounding tumor microenvironment, thereby contributing to the high vascularization and invasive properties of EWS [116,119,122]. Validation of the EWSR1-ETS upregulated transcription factors NKX2.2, NR0B1, GLI1, BCL11B and E2F3 demonstrated an extensive attribution to the aggressive and stem-like phenotype of EWS [115,123,124,125,126]. EWSR1-ETS affects gene expression, mainly downregulation, both directly and indirectly. Directly, by binding GGAA microsatellites and indirectly, by interacting with the NuRD co-repressor complex and upregulating above mentioned transcription factors [101,115,125]. Transcription initiation is commonly not regulated by one but multiple transcription factors which interact with each other. By interacting with transcription factors, such as E2F3 and Sp1, EWSR1-ETS enhances its ability to induce gene activation [35,126,127]. Although EWSR1-ETS needs variable different cellular processes for its effect at the transcriptome level, the EWSR1-ETS map was observed to be relatively stable. When comparing the transcriptomes of cell lines with tumors in a principle component analysis, only the first principle component of pathways was significantly different. The principle component consisted of tumor-microenvironment pathways in EWS tumors and metabolic pathways in cell lines [28].

EWSR1-ETS affects not only the expression of genes but also the expression of non-coding RNAs, including both micro RNAs (miRNAs) and long non-coding RNAs (lncRNAs) [22,38,128]. miRNAs are regulating more than 60% of the human genes by mainly binding at the 3’UTR of the mRNA [129]. Around 10% of the studied miRNAs are significantly affected in EWS, both in down- and upregulation [128,130,131]. Affected pathways are diverse and include important tumorigenic pathways such as IGF signaling, chromatin remodeling, pluripotency and DNA damage repair [128,131,132,133,134]. An relatively small EWS retrospective patient survival association study on miRNAs identified a survival association with increased *miRNA34a* expression [135]. *miRNA34a* is thought not to be influenced by EWSR1-FLI1 itself but its activity is regulated by TP53 and NF-κB and is associated with survival also in a retrospective glioblastoma study [136,137,138]. It regulates expression of proteins involved in growth pathway signaling, apoptosis, chromatin remodeling and genomic stress [136,137,138]. miRNA analysis at whole transcriptome scale might be successful to identify more miRNAs regulated by EWSR1-ETS or which are predictive for therapy.

Long non-coding RNAs are relatively recently discovered as functionally relevant and have functions both in epigenetic and post-translational regulations [139,140]. For example, *MALAT1* is a commonly expressed lncRNA, which is involved in angiogenesis and cell cycle progression [141,142]. Brunner *et al.* [143] studied expression of lncRNAs in a large tumor panel of both sarcomas and carcinomas including EWS. A large number of known and novel lncRNAs differentially expressed in EWS were identified, including *ALDH1L1-AS2*, *DICER1-AS1* and *LINC00277* [143]. In later research, *LINC00277* (*EWSAT1*) was the only lncRNA which was significantly overexpressed in *EWSR1-FLI1* transfected pediatric MSCs and downregulated in EWS cell lines when treated with *EWSR1-FLI1* shRNA [38]. *LNC00277* induction on its own affected expression levels of numerous genes which overlapped with EWSR1-FLI1 target genes. Its effect was established partly by interacting with the RNA binding protein HNRNPK. A number of splice variants of *LINC00277* were described of which *LINC00277-2* was dominantly expressed in EWS. The modus of action of the various splice variants is unknown until now.

After transcription, RNAs are spliced and alternative splicing increases the functional diversity of proteins and noncoding RNAs. Splicing is regulated by multiple protein complexes and by interfering in this regulation many cellular processes can potentially be affected [144]. EWSR1 is involved in one of these protein complexes as scaffold protein [145,146]. EWSR1-ETS, missing the C-terminal part of EWSR1, interferes in the EWSR1 complex mediated splicing and causes the deregulated splicing of EWSR1 complex targeting RNAs [39,146,147,148]. One of the processes interfered with by EWSR1-ETS binding that was investigated in depth is the binding to RNA helicase A (RHA). RHA has both functions in DNA and RNA unwinding and stabilization [40,149]. Especially the RNA binding of RHA was inhibited by EWSR1-ETS binding and the new EWSR1-ETS RHA complex could bind additional targets, which were enriched for transcripts involved in extracellular signaling processes [40]. The consequences at the cellular level of the splicing interference has been illustrated by the splicing of *CCDN1* (cyclin D1), a cell cycle regulator and *Vascular Endothelial Growth Factor A* (*VEGF-A*). The normal a-isoform of CCND1 is exported from the nucleus during G1 phase to stop the cell cycle but by EWSR1-ETS interference the relative quantity of the b-isoform is increased in EWS. This isoform is not exported and increases proliferation of EWS cells [150]. *VEGF-A* splicing can result in both more and less angiogenic isoforms and, by the interference of EWSR1-ETS, the equilibrium between these isoforms is shifted to the more angiogenic isoform VEGFA-165. The effect of this shift is an increase in angiogenesis, which correlates to the highly vascularized histological features of Ewing sarcoma [151]. Despite the number of fundamental studies on the mechanism affected by EWSR1-ETS, limited studies are published on RNA targets. Whole transcriptome sequencing could be used to map the RNA targets of which splicing are affected by EWSR1-ETS but this has not yet been reported. In conclusion transcriptome mapping has shown to be of high value to characterize EWS and identify potential targets and survival markers [37,38,106,135].

## 6. Understanding Therapy Sensitivity and Identifying Target Candidates Using the Ewing Sarcoma Sequencing Overview

Before the introduction of chemotherapy, the overall survival of patients with EWS was about 10% using surgery alone. Early observation showed increased radiosensitivity of Ewing sarcoma and therefore radiotherapy as monotherapy was introduced, but the majority of patients still died of metastasis within two years when only radiotherapy was used [152,153]. The introduction of systematic chemotherapy increased the overall survival from ten percent to nowadays sixty to seventy-five percent for a localized tumor at diagnosis [2,154,155]. However, when a patient has recurrent disease, which is the case in thirty percent, or presents a metastatic disease at diagnosis, the overall survival drops to ten to forty percent [45,46]. As these patients are young, longtime curing is the treatment prospective, rather than stabilizing and short-term benefit. This translates in intense treatments but, as a pay-off, these have large consequences for long-term survival of EWS patients [156]. Prognostic markers for survival or treatment sensitivity may help to personalize the treatment [157]. New treatment protocols are needed to increase the patient survival with the least long-term effect. The uncovering of the mechanism of disease specific pathways serves as the basis for the development of targeted drugs to treat patients with the highest efficacy and the least side effects. For this, an EWS OMIC overview, using the results obtained by sequencing from various sources (Table 1), could help to increase this fundamental understanding and could lead to the identification of novel therapeutic candidates for systemic, targeted and immunotherapy.

### 6.1. DNA Damage Response and Repair: Systemic and Targeted Therapy

Chemotherapy is an essential part in the treatment of EWS [154,158]. Over the past decades, the combination of chemotherapeutics, dosage and administration protocol has been adjusted to improve tumor response and reduce toxicity [155,159,160]. The present standard treatment protocol for EWS is based on combination of vincristine, doxorubicin, ifosfamide or cyclophosphamide and etoposide [2,159]. Most of these are DNA damaging drugs. As EWS contains a limited number of secondary mutations, it is likely that these tumors have an intact DNA damage response mechanism. Alkylating and double strand break causing agents consequently activate this mechanism leading to growth arrest and apoptosis of EWS cells. The hypothesis of an intact DNA damage response mechanism correlates with the chemotherapeutic resistance of *TP53* mutated EWS tumors, a key gene in this mechanism, since these tumors do not have an intact DNA damage response mechanism [41]. Fusion positive RMS has a limited number of mutations, similarly to EWS, but it is less sensitive to chemotherapy compared to translocation negative RMS [44]. This implies that downstream EWSR1-ETS effects may partly be responsible for the chemotherapy and radiotherapy sensitivity. In accordance, EWSR1-ETS associated DSBs have been identified and radiation induced damage turnover in EWS was reduced compared to osteosarcoma [161,162]. This makes the damage repair pathway a promising candidate to target. Compromising the DNA damage repair pathway via inhibition of poly (ADP ribose) polymerase 1 (PARP1) did indeed lead to inhibited proliferation in EWS cell lines and potentiated the response to temozolomide and irinotecan [19,161,162,163]. However, in EWS xenografts and patients treated with a PARP1 inhibitor, only the combination with temozolomide or irinotecan was effective [162,164]. In colon cancer xenografts this effect was observed as well [165]. The sensitivity of colon cancer to PARP1 inhibition is hypothesized to be related to a less functional homologous recombination due to cohesin complex aberrancy [75,77,166]. In glioblastoma cells, a correlation between PARP1 sensitivity and the presence or absence of the cohesin complex gene *STAG2* was demonstrated [167]. In the EWS PARP1 inhibition studies both *STAG2* wild type and mutant cell lines were sensitive to PARP1 inhibition in combination with chemotherapy [163,168]. A specific role for STAG2 in this is therefore unlikely in EWS. Overall, it seems EWSR1-ETS interferes in the DNA damage repair pathway by a yet unexplainable way based on data obtained from genome and transcriptome sequencing studies leading to chemotherapy sensitivity. Identifying the TP53 independent DNA damage response and repair pathway could open novel therapeutic options.

### 6.2. Targeting Chromatin Remodeling; EWSR1-ETS and Its Binding Partners

As mentioned, EWSR1-ETS intervenes in chromatin remodeling in multiple ways and the chromatin state around GGAA microsatellites might be related to the oncogenic capacity of EWSR1-ETS. Hence, chromatin remodeling is a good target. Understanding the action of EWSR1-ETS fusion protein in this could be used to design novel therapeutic agents that either occupies its GGAA microsatellite binding sites, targets the chromatin remodeling or blocks binding of its partners in transcription.

Chemotherapeutic drugs induce DNA damage by binding to the DNA, but the same binding can interfere with the binding of EWSR1-ETS to the DNA. Between these DNA binding chemotherapeutic agents, there is a difference in binding specificity, where Cisplatin and Doxorubicin are suggested to be less specific than Actinomycin D for removal of EWSR1-ETS from the DNA [169]. However, due to the heavy systemic side effects Actinomycin D is no longer used to treat Ewing sarcoma patients in the U.S. [170]. Trabectedin, a toxin from the sea squirt *Ecteinascidia turbinate*, is believed to be more specific against EWSR1-ETS DNA binding sites. *In vitro* studies in EWS and myxoid liposarcoma, another fusion gene holding tumor demonstrated a high efficacy and showed interference with the activity of EWSR1-ETS and EWSR1-CHOP fusion protein, respectively [158,171]. In a clinical trial, however, trabectedin alone did not show a significant effect on overall survival in EWS [172].

Targeting chromatin remodelers, for example LSD1 and HDAC2, that attribute to the EWSR1-ETS oncogenic potential has been shown to be effective *in vitro* and in xenografts [37,106,173,174]. The effect of inhibiting LSD1 was even analyzed by whole transcriptome sequencing in cell lines with *EWSR1-FLI1* or *EWSR1-ERG* translocation to identify the overall effect on gene expression. Inhibition affected numerous genes including well-known target genes like *CAV1*, *NKX2.2*. This inhibition study strengthened the role of the NuRD complex in the transcriptome wide effect of EWSR1-ETS [37]. HDAC2 inhibition by Vorinostat had a similar effect on the EWSR1-ETS repressed genes, replicating an earlier HDAC2 inhibition study in EWS, but did not affect genes directly activated by EWSR1-ETS [37,175]. A third potential identified target is the EWSR1-ETS upregulated histone deacetylase *SIRT1* which was identified in a *EWSR1-FLI1* knock-down screen and inhibition was effective in EWS cell lines *in vitro* and in xenografts [106]. Furthermore, *SIRT1* is regulated by miRNA34a expression, a prognostic factor in EWS, and both are associated with TP53 activity [135,137].

Riggi *et al.* [31] demonstrated that EWSR1-ETS can function as initiator in chromatin remodeling but needs to recruit other proteins for transcription initiation. Agents that inhibit the interaction between EWSR1-ETS and its binding partners by blocking the binding sites of EWSR1-ETS can be fruitful and a daunting task at the same time due to the disordered structure of EWSR1-ETS [176]. The identification of the small molecule YK-4-279 as inhibitor of the EWSR1-ETS binding to RHA confirmed that this approach is indeed promising and had a broad transcriptomic influence in EWS [40,177,178]. YK-4-279 treatment resulted in the same effect at splicing level as *EWSR1-ETS* inhibition and inhibited the binding of transcripts by the EWSR1-ETS RHA complex [39,40]. *In vivo* experiments suggest that combining this agent with other treatments could be especially effective, as shown in the combination with TP53 reactivating agent Nutlin3a in a zebrafish model [179].

### 6.3. Targeting EWSR1-ETS Influenced Extracellular Signaling, Transcriptome Mapping as a Lead

At the transcriptome level, EWSR1-ETS influences various pathways involved in intracellular processes and tumor microenvironmental processes that are needed for EWS development and maintenance. Both these processes are vital according to the processes collectively described as the hallmarks of cancer by Hanahan and Weinberg [180]. Major pathways by EWSR1-ETS affected are involved in extracellular signaling and membrane protein signaling. At a histo-morphological level, this is reflected by a stem-cell like tumor with high vascularization and a clinically observed high metastatic potential. Involved EWSR1-ETS key target pathways responsible for these features might be identified by transcriptome sequencing.

A well-known EWSR1-ETS targeted pathway is the IGF pathway, which is involved in tumor growth, metastasis and angiogenesis [181,182]. EWSR1-ETS increases the IGF1 pathway activity by upregulation of IGF1 expression and downregulation of insulin growth factor binding protein 3 and 5 (IGFBP3, IGFBP5) and various IGF pathway targeting miRNAs [114,117,133]. Targeting this pathway by small molecules or by monoclonal antibodies was shown to be highly effective in cell lines and it inhibited the angiogenesis in xenografts. In clinical trials, IGF1R treatment resulted in partial success due to no-response or quick resistance while a small group of patients remained stable. By studying the long-term responding patients at OMIC levels, we could identify the cause of their tumors sensitivity to anti-IGF therapy, which, in turn, would identify patients for anti-IGF therapy and understand the mechanism of the gained resistance [183,184,185,186,187]. In addition, combination chemotherapy with anti-IGF therapy is being investigated and may be an option. The combination of OSI-906, a dual inhibitor of IGF1R and IR, with trabectedin showed promising preclinical results [188].

The introduction of anti-angiogenic therapy with promises for all cancer types has been taking full media coverage with high initial expectations. Massive efforts to develop novel anti-angiogenic agents have led to several novel targeted therapy approaches. Although anti-angiogenic therapy alone was found to be insufficient, a combination with other treatment modalities may be effective [189]. As Ewing sarcoma is highly vascularized, targeting angiogenesis has been investigated in several *in vitro* and *in vivo* studies with success [185,190,191,192]. This was translated into multiple clinical trials testing anti-angiogenic drugs (NCT00516295, NCT01946529, NCT01492673 and NCT02243605). However, a pilot study in which chemotherapy with or without bevacizumab (a VEGF-A inhibitor) was used did not show a positive effect of bevacizumab [193]. Vascular mimicry has been observed in Ewing sarcoma and may be enhanced under hypoxic conditions that might reverse the anti-angiogenic effect [194,195,196].

As was demonstrated by micro-array studies and verified by transcriptome sequencing, EWSR1-ETS represses extracellular signaling proteins, including chemokines [28,38,113]. Chemokines are involved in all important tumor microenvironmental processes and elucidating the relation between the presence or absence of these chemokines may lead to new candidate targets or reactivating agents which would increase the chemokine expression [197]. The expression levels of the pro-inflammatory chemokines CXCL9 and 10 have been linked to the number of infiltrating T-cells and subsequently with a better overall survival in EWS patients in a relatively small retrospective study in a univariate analysis [198]. Treatment with interferon gamma (IFN-ƴ) enhanced the expression levels of pro-inflammatory chemokines and sensitizes resistant EWS cells *in vitro* to tumor necrosis factor apoptosis-inducing ligand (TRAIL)-induced apoptosis [198,199]. If these processes are related to each other is unknown. The only chemokine receptor which is highly expressed in EWS and not repressed by EWSR1-ETS is CXCR4 [38,200]. As a key factor in the tumor-microenvironment processes, especially metastasis, it is a very interesting receptor to study in EWS as a potential biomarker and therapeutic candidate [201,202]. Its RNA expression was, like many other tumors including osteosarcoma, correlated with lung metastasis and *in vitro* membrane CXCR4 positive cell lines migrated towards a CXCL12 gradient [200,203,204]. In contrast, no CXCR4 was detected at protein level in EWS lung metastases with immunohistochemistry but was positive in the chemotherapy-naïve tumor biopsies where it correlated with tumor volume [205]. The cause of the contradiction is unknown up to now and may be attributed to different CXCR4 isoforms or the abundant post-transcriptional modifications of CXCR4 [206,207,208,209,210].

The mentioned pathways are just examples of the many candidate pathways which can be targeted in EWS. These candidate pathways connect intracellular processes with interactions in the microenvironment and can therefore be ideal for combined therapy. OMICs can contribute to identify targets at DNA and RNA level, but since these pathways are highly interconnected with each other additional post-transcriptional studies are needed for target validation and understanding the role of these signaling pathways in EWS. An example of such a post-transcriptional study is a knockdown study [211].

### 6.4. Targeting EWS with Immunotherapy

Immunotherapy is based on the use of two general mechanisms: (1) activating the native immune system (2) priming natural killer (NK) cells or cytotoxic T-cells for antigens specifically overexpressed in the tumor to treat. The performed OMIC studies in EWS can be of value in both cases. Expression of antigen presenting and NK cell ligands in EWS samples can be determined retrospectively and EWS specific antigens can be identified.

Determination of the tumor-associated leukocytes in pediatric tumors showed an increase in macrophages and almost lack of dendritic compare to adult tumors as a common feature the almost lack of dendritic cells [212]. The determination of the presence of intratumoral leukocytes is particularly important since high numbers of CD8+ T-cells have retrospectively been found to be associated with improved survival in an univariate analysis [198]. Indirect attraction of these CD8+ T-cells to EWS may be enhanced by the IFN-ƴ therapy, since it upregulates pro-inflammatory chemokine expression levels [198]. Activation and attraction of the T-cells is presently not tested in a clinical trial but activation of endogenous or donor NK cells has been shown to be effective in EWS and phase I and II clinical trials and are currently open for enrollment (NCT01287104, NCT02100891) [213,214]. The efficacy of recognition may in fact be increased by combining this with the earlier mentioned chromatin remodeler inhibitors. *In vitro* HDAC inhibitor enhanced the NKGD ligands expression in EWS cell lines, which are essential for NK mediated lysis [215]. For improved long-term NK activation and to overcome tumor mediated downregulation of NKGD, prolonged *ex vivo* activation or antibody dependent cytolysis is needed [216]. From a preclinical perspective, allografting may be a beneficial adjuvant therapy in combination with either EWSR1-ETS blocking therapy or standard chemotherapy.

The second general method of priming cytotoxic T-cells for tumor specific membrane proteins overexpressed by EWS or unique HLA presented peptides has been investigated. Proposed targets are the tumor specific membrane proteins like PRAME, GPR64 and STEAP1 [217,218,219]. However, *ex vivo* priming of T-cells for antigens like PRAME and STEAP1 could not yet induce a prolonged antitumor immune response in preclinical studies. T-cells could not interact with the endogenous presented antigens at EWS cell lines or the T-cells which did recognize the presented antigens were classified as exhausted T-cells according to high PD1 expression [220,221]. The EWSR1-ETS upregulated proteins EZH2 and CHM1 were successfully used to prime allo-restricted T-cells but these are proteins expressed in many other tissues and could have serious side effects and lead to non-tumor specific targeting [221]. The ideal antigen to prime T-cells for would be EWSR1-ETS itself. The potential of this hypothesis was tested and a EWSR1-FLI1 specific antigen was identified and verified as an activating antigen for cytotoxic T-cells, but no follow-up study has been presented [222]. A possible cause for the less effective recognition demonstrated in EWS cell lines, and the potential clinical limiting factor, is the loss of major histocompatibility complex (MHC) class I and class II, which are needed for a proper immune response [223]. Genetic engineering of T-cells with chimeric antigen receptors directed to overexpressed surface markers is independent of the MHC class system. These can be designed against proteins and even phosphoglycolipids [224]. The singular tested surface marker with this method is the neural ganglioside G_D2_ which was expressed in all 10 EWS cell lines tested and 12 of 14 analyzed patients [225]. A follow-up xenograft study was successful showing a reduction in tumor growth and number of tumors but effect on total survival was not significantly different [224]. For further research, antigens should be selected for EWS specific surface markers to prevent non-tumor cells to be targeted, like stem cells which do not express MHC class II complex but do express certain surface markers [226]. For example, the aforementioned G_D2_ is also expressed in neural crest cells and mesenchymal stem cells [227].

Overall, immunotherapy can be promising in combination with systemic or targeted therapy. Both NK-cell and T-cell related therapies have potential, especially the antibody targeted NK-cells and genetically modified T-cells. Genetically modified T-cell therapy in EWS is just starting and all membrane proteins and potential tumor specific splice variants of membrane proteins could be targets for these T-cells. In addition, the ability to target glycolipid structures opens a complete new set of possibilities, but these cannot be identified by sequencing.

## 7. Conclusions

By sequencing EWS at the genome, epigenome and transcriptome level, an atlas can be created which would help to fundamentally understand EWS and help to identify important nodes as therapeutic candidates. The *EWSR1-ETS* translocation is the characteristic pathognomonic alteration found in all tumors so far. The fusion protein act as a strong transforming oncogene and, in experimental conditions, the transfection of cells with normal cellular backgrounds rather leads to oncogene-induced apoptosis than to transformation. Recent studies, however, showed that stem cells from young individuals with the necessary permissive background did form tumors, pointing towards the importance of epigenetic controlling in cellular/tissue differentiation in providing the necessary niche for the transformation [19,22,25,38]. Therefore, mapping these genomic and functional genomic alterations can lead to identification of the cell of origin, improvements in prediction of clinical outcome, and discovery of novel therapeutic targets. These prospects have led to numerous, independent investigations using various approaches related to OMICs.

As a result, several novel findings and confirmations of earlier observations were collected. For example, some secondary structural alterations can be detected in a subset of the tumors that can identify a patient with unfavorable prognosis. Despite huge efforts to identify secondary mutations that provide a permissive background to transform cells with the pathognomonic EWSR1-ETS translocation, only a limited number of secondary point mutations were detected in EWS. Of these, *STAG2* and *TP53* were the most frequently mutated genes and mutations in these genes were associated with inferior prognosis. The value of these mutations as prognostic markers has to be validated in a prospective study. Transcriptome sequencing projects excluded the possibility of recurrent co-occurring fusion genes that would be responsible for the transformation to endure the fusion protein. Based on these massive sequencing efforts, it is likely that the EWSR1-ETS fusion can propagate transformation in cells with less differentiated features and the epigenetic landscape of these primitive cells form a permissive niche for oncogenic transformation. EWSR1-ETS is, both at the transcriptome and epigenome level, the most dominant actor both by activation and repression transcription and needs cooperation of binding partner proteins like chromatin remodelers. The identified key pathways in Ewing sarcoma and the EWSR1-ETS chromatin remodeling binding partners include promising candidate targets. This needs to be validated with in functional studies in combination with the epigenome and transcriptome analyses.

The advantage of the genomic stability of EWS is that the endogenous pathways controlling DNA damage recognition and apoptosis are still intact and could potentially be activated when targeted specifically and especially together with agents acting on the basis of the EWSR1-ETS network of epigenomic and transcriptomic changes. For example, blocking the interaction with its binding proteins could be a very efficient combination therapy. By deciphering this network for both targeted therapy as well as immunotherapy, novel key target candidates can be identified. In the future, hopefully these therapies could, together with conventional chemotherapy, improve the outcome of these young patients.
